# Extracorporeal co2 removal in hypercapnic patients who fail noninvasive ventilation and refuse endotracheal intubation: a case series

**DOI:** 10.1186/2197-425X-3-S1-A824

**Published:** 2015-10-01

**Authors:** A Morelli, A D'Egidio, A Orecchioni, F Alessandri, L Mascia, VM Ranieri

**Affiliations:** Policlinico Umberto I° University of Rome La Sapienza, Anesthesiology and Intensive Care, Rome, Italy

## Introduction

Noninvasive ventilation (NIV) represents the standard of care for patients with exacerbation of chronic obstructive pulmonary disease. However, NIV fails in almost 40% of the most severe forms of acute hypercapnic respiratory failure and patients must undergo endotracheal intubation and invasive ventilation. Such transition from NIV to invasive ventilation is associated to increased mortality. Under these circumstances, patients may express a clear intention not to be intubated.

## Objectives

To assess efficacy and safety of noninvasive ventilation- plus-extracorporeal Co2 removal in patients who fail NIV and refuse endotracheal intubation.

## Methods

We reported data from a case series of 30 patients with acute hypercapnic respiratory failure due to exacerbation of chronic obstructive pulmonary disease, who refused endotracheal intubation after failing NIV and therefore were treated with extracorporeal Co2 removal plus NIV as last resort therapy. All patients acknowledged the nature of last resort therapy and gave consent to treatment. Collected data of these patients were then retrospectively matched with data obtained from 30 historical controls who received conventional treatment with endotracheal intubation.

## Results

**Figure 1:**
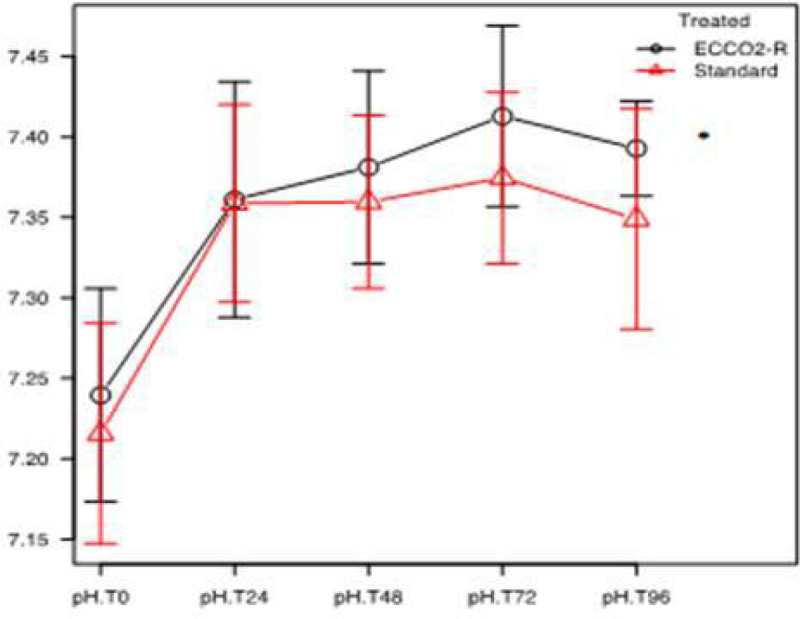
Figure 1

**Figure 2:**
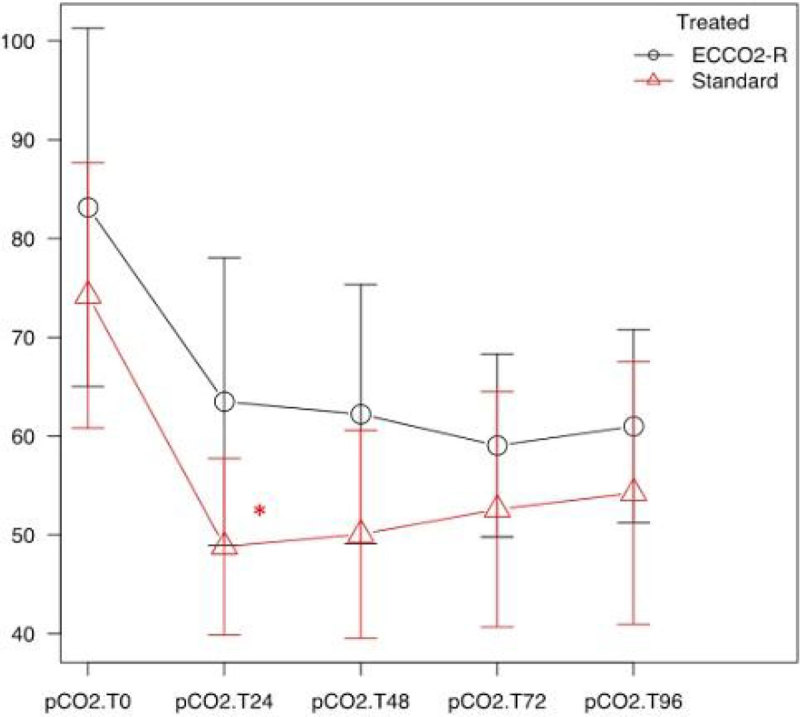
Figure 2

**Figure 3:**
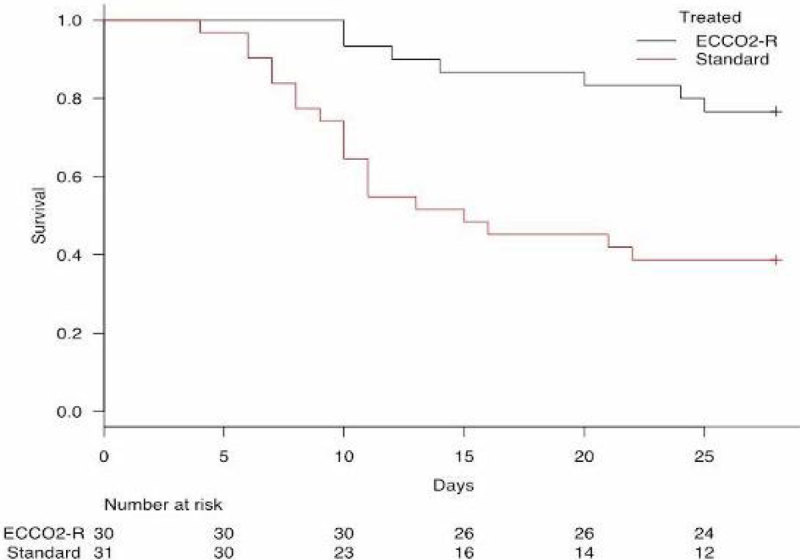
Figure 3

After matching the patients, demographic characteristics including age, BMI, gender and SOFA II score did not differ between the two study groups. At the baseline, 20 patients in the treated group and 16 in the control group required norepinephrine to maintain mean arterial pressure, at the doses of 0.46 ± 0.18 and 0.37 ± 0.15 (mean ± SD) µg/Kg/min respectively, without statistically significant differences.A significant reduction of PaCo2 from baseline to 96 hours of treatment was observed in both group (p < 0.05) and reached between groups difference only at 24 hours. An increase in arterial pH were observed from baseline to 96 hours of treatment in both groups (p < 0.05) and between groups difference was observed at 96 hours (p < 0.004, Fig 1 and 2). The duration of extracorporeal Co2 removal was 4.8 ± 3 (mean ± SD) days. The longer duration of treatment was 16 days. At day 14, the percentage of patients requiring norepinephrine was lower in the treated group compared to the control group, 30 % vs. 60 % respectively (p = 0.04). Mortality at day 28 was significantly lower in the treated group than in control group (23.3 % vs. 58.1 %, p < 0.001, Fig 3). In the treated group none of patients experienced bleeding events with a heparin infusion in the circuit of 5.6 ± 1.5 (mean ± SD) UI/Kg/h. Nevertheless 8 patients had clots in the circuit which required the substitution of the circuit.

## Conclusions

Our results support the need for a large randomized controlled clinical trial to test the hypothesis that extracorporeal Co2 removal may contribute to improve survival in patients with acute hypercapnic respiratory failure due to exacerbation of chronic obstructive pulmonary disease.

